# Barium appendicitis

**DOI:** 10.1002/ccr3.4583

**Published:** 2021-07-23

**Authors:** Daisuke Omura, Naoko Yunoki, Yuki Otsuka, Yasuhiro Nakano, Hideharu Hagiya, Fumio Otsuka

**Affiliations:** ^1^ Department of General Medicine Dentistry and Pharmaceutical Sciences Okayama University Graduate School of Medicine Okayama Japan; ^2^ Department of Internal Medicine Akaiwa Medical Association Hospital Akaiwa Japan

**Keywords:** appendicitis, barium

## Abstract

We have presented a case of barium appendicitis, which is a rare complication of barium enema studies. Barium sulfate is used widely for gastrointestinal radiographic studies and is associated with few complications. Clinicians need to be fully aware of this complication.

## Graphical abstract

We have presented a case of barium appendicitis, which is a rare complication of barium enema studies. Barium sulfate is used widely for gastrointestinal radiographic studies and is associated with few complications. Clinicians need to be fully aware of this complication. (41 words).

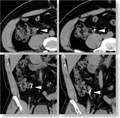

A previously healthy 42‐year‐old man was admitted to our hospital with a complaint of abdominal pain for a day. The patient's vital signs were within normal range, and he had no physical findings other than tenderness in the upper right abdomen. Blood analysis showed elevations of C‐reactive protein (5.4 mg/dl). Computed tomography revealed high density in the appendix (Figure 1), and the patient was diagnosed with appendicitis. Subsequent interviews revealed that the patient was taking barium for a medical examination 3 days before his visit. The patient underwent antibiotic treatment for barium appendicitis. The patient had no peritoneal irritation symptoms and responded only to medical therapy.

**FIGURE 1:**
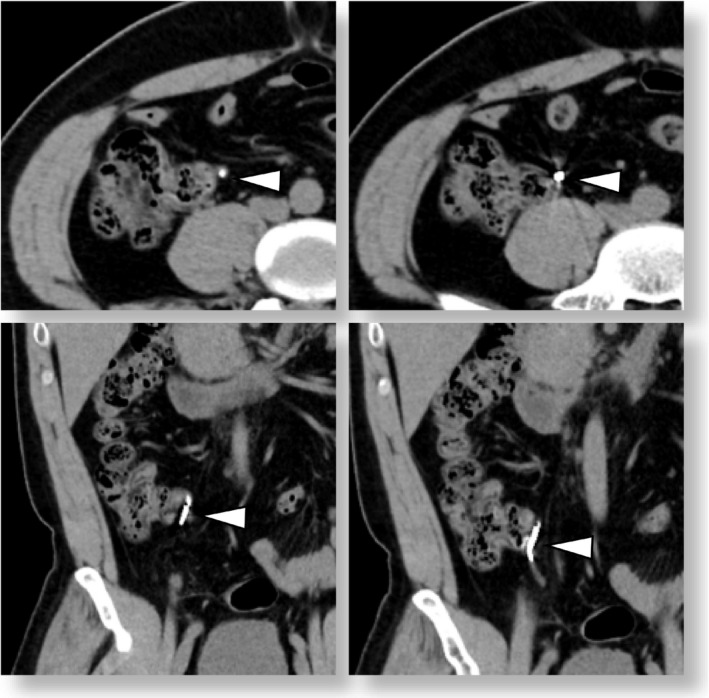
Abdominal computed tomography

Barium remains in the appendix for more than 72 h in only 8% of patients,^1^ and in a small number of these patients, the remaining barium obstructs the lumen of the appendix and causes appendicitis. The prognosis and treatment of patients with barium appendicitis are not particularly different from those of patients with non‐barium appendicitis. However, the frequency of appendiceal perforation in barium appendicitis was reported to be higher (not significantly) than that in non‐barium appendicitis.^2^ Since barium is widely used in gastric cancer screening in Japan, the diagnosis and recognition of barium appendicitis are particularly valuable.

## CONFLICT OF INTEREST

None declared.

## AUTHOR CONTRIBUTIONS

DO wrote the first draft and managed all of the submission process. NY, YO, and YN supervised clinical management of the patient. HH and FO contributed to clinical management of the patient and revised the manuscript.

## ETHICAL APPROVAL

Informed consent was obtained from the patient to publish this case report.

## Data Availability

Not applicable.
